# Crystal structure of 3-(2-nitro­phen­yl)-1-(1-phenyl­sulfonyl-1*H*-indol-3-yl)propan-1-one

**DOI:** 10.1107/S2056989015020162

**Published:** 2015-10-31

**Authors:** M. Umadevi, Potharaju Raju, R. Yamuna, Arasambattu K. Mohanakrishnan, G. Chakkaravarthi

**Affiliations:** a Research and Development Centre, Bharathiar University, Coimbatore 641 046, India; b Department of Chemistry, Pallavan College of Engineering, Kanchipuram 631 502, India; c Department of Organic Chemistry, University of Madras, Guindy Campus, Chennai 600 025, India; d Department of Sciences, Chemistry and Materials Research Lab, Amrita Vishwa Vidyapeetham University, Ettimadai, Coimbatore 641 112, India; e Department of Physics, CPCL Polytechnic College, Chennai 600 068, India

**Keywords:** crystal structure, indole, hydrogen bonding, C—H⋯π inter­actions

## Abstract

In the title compound, C_23_H_18_N_2_O_5_S, the phenyl and benzene rings subtend dihedral angles of 78.18 (10) and 30.18 (9)°, respectively, with the indole ring system (r.m.s. deviation = 0.022 Å). The crystal structure features weak C—H⋯O and C—H⋯π inter­actions, which link the mol­ecules into a three-dimensional network.

## Related literature   

For the biological activity of indole derivatives, see: Andreev *et al.* (2015 ▸); Kolocouris *et al.* (1994 ▸). For related structures, see: Chakkaravarthi *et al.* (2007 ▸, 2008 ▸).
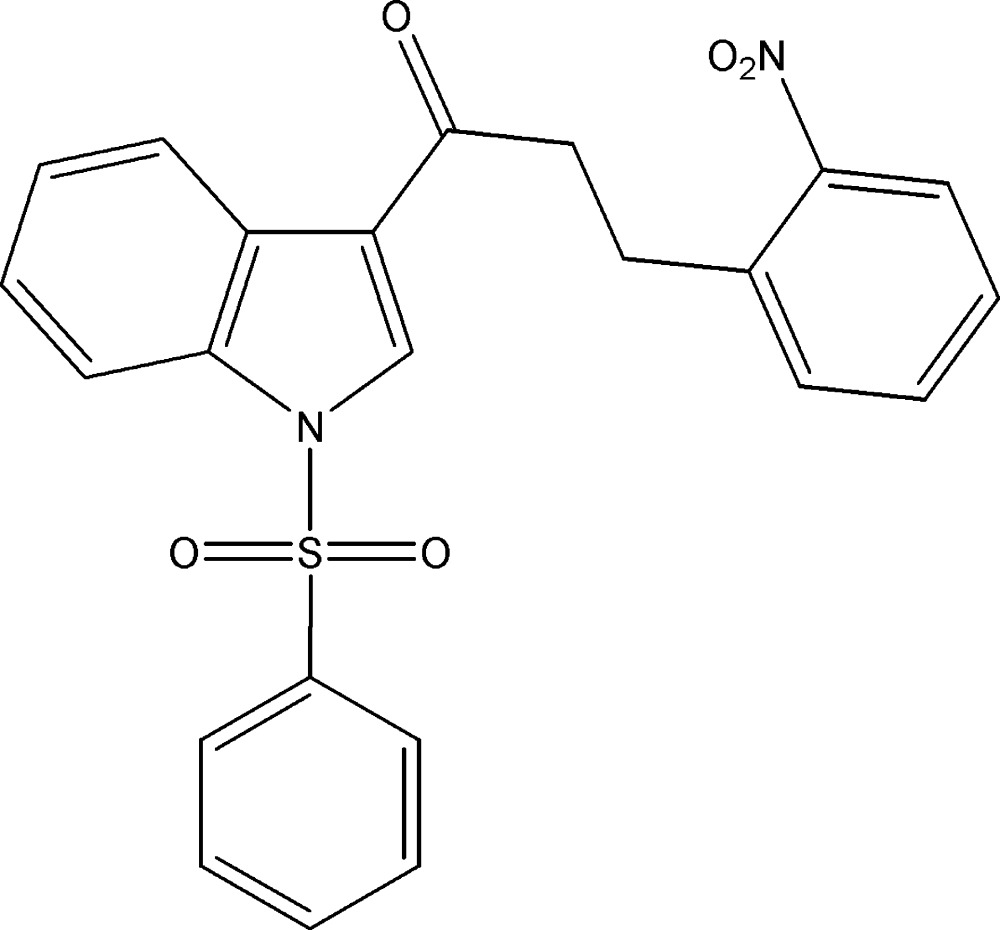


## Experimental   

### Crystal data  

C_23_H_18_N_2_O_5_S
*M* *_r_* = 434.45
Monoclinic, 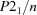

*a* = 9.0224 (7) Å
*b* = 15.4581 (10) Å
*c* = 15.1347 (10) Å
β = 106.349 (2)°
*V* = 2025.5 (2) Å^3^
*Z* = 4
Mo *K*α radiation
μ = 0.20 mm^−1^
*T* = 295 K
0.26 × 0.24 × 0.20 mm

### Data collection  

Bruker Kappa APEXII CCD diffractometer
Absorption correction: multi-scan (*SADABS*; Sheldrick, 1996 ▸) *T*_min_ = 0.950, *T*_max_ = 0.961
29555 measured reflections
6149 independent reflections
4060 reflections with *I* > 2σ(*I*)
*R*_int_ = 0.033

### Refinement  

*R*[*F* ^2^ > 2σ(*F* ^2^)] = 0.058
*wR*(*F* ^2^) = 0.144
*S* = 1.09
6149 reflections
280 parameters
3 restraints
H-atom parameters constrained
Δρ_max_ = 0.35 e Å^−3^
Δρ_min_ = −0.32 e Å^−3^

### 

Data collection: *APEX2* (Bruker, 2004 ▸); cell refinement: *SAINT* (Bruker, 2004 ▸); data reduction: *SAINT*; program(s) used to solve structure: *SHELXS97* (Sheldrick, 2008 ▸); program(s) used to refine structure: *SHELXL97* (Sheldrick, 2008 ▸); molecular graphics: *PLATON* (Spek, 2009 ▸); software used to prepare material for publication: *SHELXL97* and *PLATON*.

## Supplementary Material

Crystal structure: contains datablock(s) global, I. DOI: 10.1107/S2056989015020162/hb7524sup1.cif

Structure factors: contains datablock(s) I. DOI: 10.1107/S2056989015020162/hb7524Isup2.hkl

Supporting information file. DOI: 10.1107/S2056989015020162/hb7524Isup3.cml

**Fig. 1.**
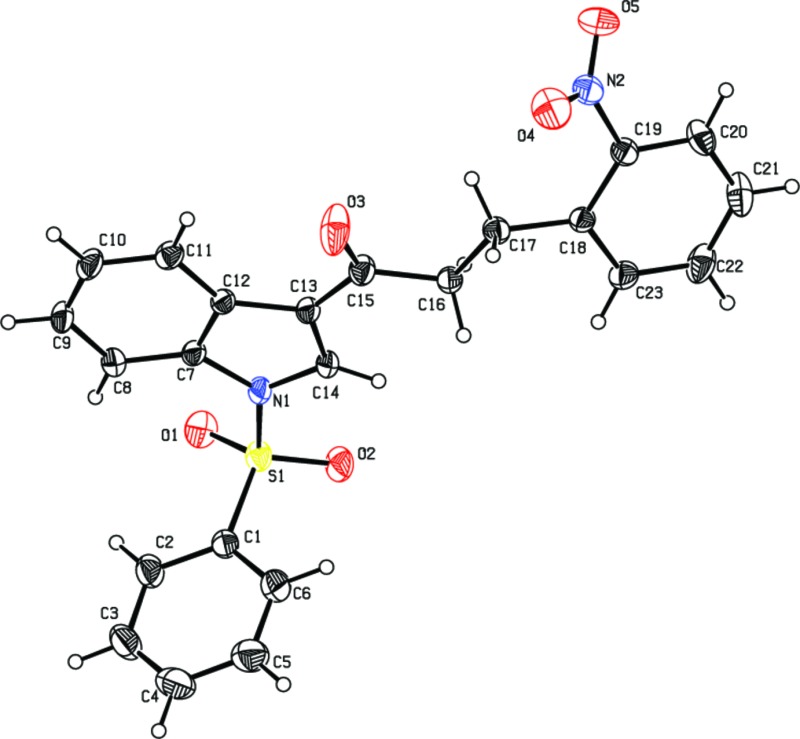
The molecular structure of the title compound, with displacement ellipsoids drawn at the 30% probability level.

. DOI: 10.1107/S2056989015020162/hb7524fig1.tif
The mol­ecular structure of the title compound, with displacement ellipsoids drawn at the 30% probability level.

**Fig. 2.**
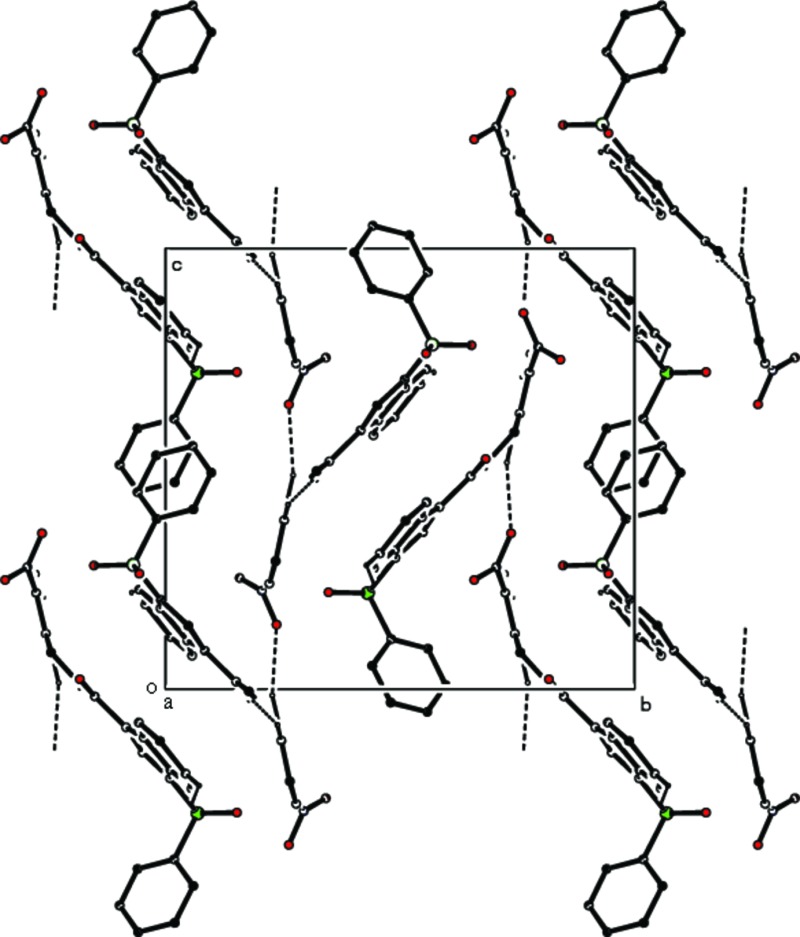
The crystal packing of the title compound, viewed along the a axis. The C—H···O hydrogen bonds are shown as dashed lines (see Table 1). H atoms not involved in these interactions have been omitted for clarity.

. DOI: 10.1107/S2056989015020162/hb7524fig2.tif
The crystal packing of the title compound, viewed along the a axis. The C—H⋯O hydrogen bonds are shown as dashed lines (see Table 1). H atoms not involved in these inter­actions have been omitted for clarity.

CCDC reference: 1433093

Additional supporting information:  crystallographic information; 3D view; checkCIF report

## Figures and Tables

**Table 1:** Hydrogen-bond geometry (, ) *Cg*3 is the centroid of the C7C12 ring.

*D*H*A*	*D*H	H*A*	*D* *A*	*D*H*A*
C9H9O2^i^	0.93	2.54	3.328(2)	143
C22H22O3^ii^	0.93	2.42	3.319(3)	163
C23H23O5^iii^	0.93	2.36	3.247(3)	160
C16H16*A* *Cg*3^iv^	0.97	2.73	3.565(2)	144

Symmetry codes: (i) 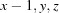; (ii) 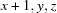; (iii) 
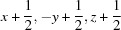
; (iv) 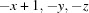.

## supplementary crystallographic information

### S1. Structural commentary

Indole derivatives are known to exhibit anti-hepatitis C virus (Andreev *et al.*, 2015) and anti­viral activity (Kolocouris *et al.*, 1994). We herein report the crystal structure of (I) (Fig. 1). The *ORTEP* diagram of the title compound (I) is shown in Fig. 1. The geometric parameters of (I) are comparable with similar structures (Chakkaravarthi *et al.* 2007, 2008).

The phenyl ring (C1—C6) and benzene ring (C18—C23) make the dihedral angles of 78.18 (10)° and 30.18 (9)°, respectively with the indole ring system. The phenyl (C1—C6) and benzene (C18—C23) rings are inclined at angle of 69.93 (12)°. In the crystal structure, the inter­molecular weak C—H···O and C—H···π (Fig. 2 & Table 1) inter­actions form a three dimensional network.

### S2. Synthesis and crystallization

To a solution of 1-(phenyl­sulfonyl)-1H-indole (0.5 g, 1.94 mmol) in dry DCM 3-(2-nitro­phenyl)­propanoyl chloride (0.62 g, 2.92 mmol) and SnCl_4_ (0.76 g, 2.92 mmol) were added slowly at 273 K under nitro­gen atmosphere and the resulting mixture was stirred at room temperature for 30 min after completion of starting material (monitored by TLC), the reaction mass was poured over ice water containing Conc. HCl (3 ml) and extracted with DCM (20 ml). The combined organic extracts were washed with water (30 ml), brine solution (10 ml) and dried Na_2_SO_4_. Removal of solvent followed by recrystallization of the crude product from methanol (3 ml) solution afforded the title compound as colourless blocks.

### S3. Refinement

H atoms were positioned geometrically and refined using riding model, with C—H = 0.93 Å and *U*_iso_(H) = 1.2Ueq(C) for C—H and C—H = 0.97 Å and *U*_iso_(H) = 1.2Ueq(C) for CH_2_. The reflection (0 1 1) is omitted during refinement which is owing poor agreement.

### Crystal data

**Table:**

C_23_H_18_N_2_O_5_S	*F*(000) = 904
*M**_r_* = 434.45	*D*_x_ = 1.425 Mg m^−^^3^
Monoclinic, *P*2_1_/*n*	Mo *K*α radiation, λ = 0.71073 Å
Hall symbol: -P 2yn	Cell parameters from 7714 reflections
*a* = 9.0224 (7) Å	θ = 2.4–28.3°
*b* = 15.4581 (10) Å	µ = 0.20 mm^−^^1^
*c* = 15.1347 (10) Å	*T* = 295 K
β = 106.349 (2)°	Block, colourless
*V* = 2025.5 (2) Å^3^	0.26 × 0.24 × 0.20 mm
*Z* = 4	

### Data collection

**Table:**

Bruker Kappa APEXII CCD diffractometer	6149 independent reflections
Radiation source: fine-focus sealed tube	4060 reflections with *I* > 2σ(*I*)
Graphite monochromator	*R*_int_ = 0.033
ω and φ scan	θ_max_ = 31.6°, θ_min_ = 2.4°
Absorption correction: multi-scan (*SADABS*; Sheldrick, 1996)	*h* = −12→11
*T*_min_ = 0.950, *T*_max_ = 0.961	*k* = −21→22
29555 measured reflections	*l* = −21→20

### Refinement

**Table:**

Refinement on *F*^2^	Primary atom site location: structure-invariant direct methods
Least-squares matrix: full	Secondary atom site location: difference Fourier map
*R*[*F*^2^ > 2σ(*F*^2^)] = 0.058	Hydrogen site location: inferred from neighbouring sites
*wR*(*F*^2^) = 0.144	H-atom parameters constrained
*S* = 1.09	*w* = 1/[σ^2^(*F*_o_^2^) + (0.048*P*)^2^ + 1.0896*P*] where *P* = (*F*_o_^2^ + 2*F*_c_^2^)/3
6149 reflections	(Δ/σ)_max_ < 0.001
280 parameters	Δρ_max_ = 0.35 e Å^−^^3^
3 restraints	Δρ_min_ = −0.32 e Å^−^^3^

### Special details

**Table:**

Geometry. All esds (except the esd in the dihedral angle between two l.s. planes) are estimated using the full covariance matrix. The cell esds are taken into account individually in the estimation of esds in distances, angles and torsion angles; correlations between esds in cell parameters are only used when they are defined by crystal symmetry. An approximate (isotropic) treatment of cell esds is used for estimating esds involving l.s. planes.
Refinement. Refinement of F^2^ against ALL reflections. The weighted R-factor wR and goodness of fit S are based on F^2^, conventional R-factors R are based on F, with F set to zero for negative F^2^. The threshold expression of F^2^ > 2sigma(F^2^) is used only for calculating R-factors(gt) etc. and is not relevant to the choice of reflections for refinement. R-factors based on F^2^ are statistically about twice as large as those based on F, and R- factors based on ALL data will be even larger.

### Fractional atomic coordinates and isotropic or equivalent isotropic displacement parameters (Å^2^)

**Table:**

	*x*	*y*	*z*	*U*_iso_*/*U*_eq_	
C1	0.7579 (2)	−0.01858 (14)	0.38658 (13)	0.0384 (4)	
C2	0.6908 (3)	−0.05928 (17)	0.44691 (16)	0.0514 (6)	
H2	0.6403	−0.1120	0.4318	0.062*	
C3	0.7005 (3)	−0.0197 (2)	0.53057 (17)	0.0663 (8)	
H3	0.6565	−0.0460	0.5724	0.080*	
C4	0.7746 (3)	0.0580 (2)	0.55179 (18)	0.0690 (8)	
H4	0.7801	0.0842	0.6079	0.083*	
C5	0.8408 (3)	0.09752 (19)	0.49126 (18)	0.0655 (7)	
H5	0.8907	0.1504	0.5065	0.079*	
C6	0.8337 (3)	0.05935 (16)	0.40815 (16)	0.0527 (6)	
H6	0.8793	0.0857	0.3671	0.063*	
C7	0.4643 (2)	−0.01056 (13)	0.18344 (12)	0.0327 (4)	
C8	0.3702 (2)	−0.05590 (14)	0.22590 (14)	0.0400 (5)	
H8	0.4111	−0.0932	0.2749	0.048*	
C9	0.2134 (2)	−0.04298 (15)	0.19192 (15)	0.0445 (5)	
H9	0.1467	−0.0715	0.2191	0.053*	
C10	0.1528 (2)	0.01177 (15)	0.11793 (15)	0.0445 (5)	
H10	0.0463	0.0184	0.0961	0.053*	
C11	0.2462 (2)	0.05662 (13)	0.07591 (14)	0.0390 (4)	
H11	0.2039	0.0927	0.0260	0.047*	
C12	0.4060 (2)	0.04658 (12)	0.11020 (12)	0.0314 (4)	
C13	0.5371 (2)	0.08392 (13)	0.08696 (13)	0.0330 (4)	
C14	0.6664 (2)	0.04867 (13)	0.14399 (13)	0.0349 (4)	
H14	0.7669	0.0618	0.1438	0.042*	
C15	0.5289 (2)	0.15065 (14)	0.01681 (14)	0.0407 (5)	
C16	0.6745 (2)	0.17783 (14)	−0.00516 (13)	0.0365 (4)	
H16A	0.7236	0.1273	−0.0225	0.044*	
H16B	0.7453	0.2027	0.0494	0.044*	
C17	0.6424 (2)	0.24371 (14)	−0.08307 (13)	0.0375 (4)	
H17A	0.5508	0.2259	−0.1306	0.045*	
H17B	0.6201	0.2991	−0.0596	0.045*	
C18	0.7729 (2)	0.25523 (12)	−0.12581 (12)	0.0331 (4)	
C19	0.7489 (2)	0.27562 (13)	−0.21829 (13)	0.0375 (4)	
C20	0.8658 (3)	0.27853 (15)	−0.26069 (16)	0.0512 (6)	
H20	0.8433	0.2895	−0.3235	0.061*	
C21	1.0150 (3)	0.26509 (17)	−0.2093 (2)	0.0612 (7)	
H21	1.0954	0.2681	−0.2364	0.073*	
C22	1.0444 (3)	0.24708 (17)	−0.11702 (19)	0.0583 (7)	
H22	1.1457	0.2387	−0.0814	0.070*	
C23	0.9255 (2)	0.24134 (15)	−0.07669 (15)	0.0452 (5)	
H23	0.9485	0.2277	−0.0144	0.054*	
N1	0.62637 (18)	−0.00978 (11)	0.20252 (11)	0.0368 (4)	
N2	0.5930 (2)	0.29460 (14)	−0.27675 (13)	0.0504 (4)	
O1	0.68799 (19)	−0.15240 (10)	0.28042 (12)	0.0548 (4)	
O2	0.89596 (16)	−0.05570 (11)	0.26200 (11)	0.0528 (4)	
O3	0.40514 (19)	0.18257 (14)	−0.02227 (16)	0.0843 (7)	
O4	0.5110 (2)	0.34181 (14)	−0.24777 (13)	0.0738 (6)	
O5	0.5554 (2)	0.26312 (16)	−0.35371 (12)	0.0846 (7)	
S1	0.75288 (6)	−0.06854 (4)	0.28195 (4)	0.04012 (14)	

### Atomic displacement parameters (Å^2^)

**Table:**

	*U*^11^	*U*^22^	*U*^33^	*U*^12^	*U*^13^	*U*^23^
C1	0.0297 (10)	0.0474 (12)	0.0366 (10)	0.0047 (8)	0.0069 (8)	0.0104 (9)
C2	0.0462 (13)	0.0614 (15)	0.0479 (13)	−0.0007 (11)	0.0153 (10)	0.0164 (11)
C3	0.0641 (17)	0.094 (2)	0.0461 (13)	0.0018 (16)	0.0239 (12)	0.0168 (14)
C4	0.0702 (18)	0.092 (2)	0.0437 (13)	0.0085 (16)	0.0135 (12)	−0.0041 (14)
C5	0.0732 (18)	0.0651 (17)	0.0550 (15)	−0.0096 (14)	0.0130 (13)	−0.0070 (13)
C6	0.0536 (14)	0.0578 (15)	0.0476 (13)	−0.0061 (11)	0.0155 (11)	0.0091 (11)
C7	0.0253 (9)	0.0404 (10)	0.0338 (9)	−0.0015 (7)	0.0106 (7)	−0.0059 (8)
C8	0.0353 (10)	0.0500 (12)	0.0379 (10)	−0.0038 (9)	0.0153 (8)	0.0015 (9)
C9	0.0327 (10)	0.0569 (13)	0.0499 (12)	−0.0094 (9)	0.0217 (9)	−0.0047 (10)
C10	0.0249 (9)	0.0551 (13)	0.0551 (12)	−0.0025 (9)	0.0137 (9)	−0.0089 (11)
C11	0.0297 (10)	0.0428 (11)	0.0431 (11)	0.0032 (8)	0.0078 (8)	−0.0022 (9)
C12	0.0278 (9)	0.0348 (9)	0.0327 (9)	−0.0012 (7)	0.0104 (7)	−0.0058 (7)
C13	0.0285 (9)	0.0379 (10)	0.0345 (9)	−0.0014 (7)	0.0118 (7)	−0.0030 (8)
C14	0.0262 (9)	0.0450 (11)	0.0356 (9)	−0.0024 (8)	0.0121 (7)	0.0008 (8)
C15	0.0320 (10)	0.0450 (11)	0.0458 (11)	0.0016 (9)	0.0121 (8)	0.0065 (9)
C16	0.0304 (10)	0.0447 (11)	0.0333 (9)	−0.0010 (8)	0.0071 (7)	0.0069 (8)
C17	0.0341 (10)	0.0439 (11)	0.0341 (10)	0.0030 (8)	0.0088 (8)	0.0061 (8)
C18	0.0316 (9)	0.0346 (9)	0.0312 (9)	−0.0012 (8)	0.0057 (7)	0.0035 (7)
C19	0.0391 (9)	0.0370 (10)	0.0352 (9)	0.0011 (8)	0.0084 (7)	0.0092 (8)
C20	0.0593 (15)	0.0524 (13)	0.0483 (12)	0.0049 (11)	0.0258 (11)	0.0171 (10)
C21	0.0493 (14)	0.0625 (16)	0.0833 (19)	0.0094 (12)	0.0377 (13)	0.0260 (14)
C22	0.0299 (11)	0.0644 (16)	0.0780 (17)	0.0042 (10)	0.0108 (11)	0.0224 (13)
C23	0.0353 (11)	0.0550 (13)	0.0400 (11)	−0.0012 (9)	0.0020 (8)	0.0097 (10)
N1	0.0247 (7)	0.0512 (10)	0.0353 (8)	−0.0006 (7)	0.0096 (6)	0.0061 (7)
N2	0.0463 (9)	0.0630 (12)	0.0374 (10)	0.0006 (8)	0.0042 (7)	0.0201 (9)
O1	0.0543 (10)	0.0441 (9)	0.0673 (11)	0.0027 (7)	0.0192 (8)	0.0051 (8)
O2	0.0296 (8)	0.0740 (11)	0.0580 (9)	0.0098 (7)	0.0173 (7)	0.0095 (8)
O3	0.0350 (9)	0.1001 (16)	0.1185 (17)	0.0172 (9)	0.0228 (10)	0.0669 (14)
O4	0.0556 (11)	0.0885 (14)	0.0741 (13)	0.0308 (9)	0.0130 (9)	0.0299 (10)
O5	0.0781 (14)	0.1281 (19)	0.0350 (9)	−0.0157 (13)	−0.0045 (9)	0.0106 (11)
S1	0.0301 (2)	0.0473 (3)	0.0443 (3)	0.0062 (2)	0.0126 (2)	0.0080 (2)

### Geometric parameters (Å, º)

**Table:**

C1—C6	1.378 (3)	C14—N1	1.383 (2)
C1—C2	1.381 (3)	C14—H14	0.9300
C1—S1	1.751 (2)	C15—O3	1.212 (3)
C2—C3	1.387 (4)	C15—C16	1.502 (3)
C2—H2	0.9300	C16—C17	1.523 (3)
C3—C4	1.367 (4)	C16—H16A	0.9700
C3—H3	0.9300	C16—H16B	0.9700
C4—C5	1.370 (4)	C17—C18	1.505 (3)
C4—H4	0.9300	C17—H17A	0.9700
C5—C6	1.374 (3)	C17—H17B	0.9700
C5—H5	0.9300	C18—C23	1.385 (3)
C6—H6	0.9300	C18—C19	1.391 (3)
C7—C8	1.391 (3)	C19—C20	1.381 (3)
C7—C12	1.399 (3)	C19—N2	1.465 (3)
C7—N1	1.408 (2)	C20—C21	1.368 (4)
C8—C9	1.377 (3)	C20—H20	0.9300
C8—H8	0.9300	C21—C22	1.375 (4)
C9—C10	1.387 (3)	C21—H21	0.9300
C9—H9	0.9300	C22—C23	1.379 (3)
C10—C11	1.377 (3)	C22—H22	0.9300
C10—H10	0.9300	C23—H23	0.9300
C11—C12	1.397 (3)	N1—S1	1.6736 (17)
C11—H11	0.9300	N2—O4	1.207 (3)
C12—C13	1.445 (2)	N2—O5	1.219 (3)
C13—C14	1.355 (3)	O1—S1	1.4199 (17)
C13—C15	1.467 (3)	O2—S1	1.4200 (15)
			
C6—C1—C2	121.5 (2)	C13—C15—C16	119.20 (17)
C6—C1—S1	118.98 (16)	C15—C16—C17	111.76 (16)
C2—C1—S1	119.51 (18)	C15—C16—H16A	109.3
C1—C2—C3	118.4 (2)	C17—C16—H16A	109.3
C1—C2—H2	120.8	C15—C16—H16B	109.3
C3—C2—H2	120.8	C17—C16—H16B	109.3
C4—C3—C2	120.2 (2)	H16A—C16—H16B	107.9
C4—C3—H3	119.9	C18—C17—C16	114.23 (16)
C2—C3—H3	119.9	C18—C17—H17A	108.7
C3—C4—C5	120.7 (3)	C16—C17—H17A	108.7
C3—C4—H4	119.6	C18—C17—H17B	108.7
C5—C4—H4	119.6	C16—C17—H17B	108.7
C4—C5—C6	120.2 (3)	H17A—C17—H17B	107.6
C4—C5—H5	119.9	C23—C18—C19	115.16 (18)
C6—C5—H5	119.9	C23—C18—C17	122.06 (17)
C5—C6—C1	119.0 (2)	C19—C18—C17	122.69 (17)
C5—C6—H6	120.5	C20—C19—C18	123.49 (19)
C1—C6—H6	120.5	C20—C19—N2	116.22 (18)
C8—C7—C12	122.82 (17)	C18—C19—N2	120.29 (18)
C8—C7—N1	130.07 (18)	C21—C20—C19	119.3 (2)
C12—C7—N1	107.11 (16)	C21—C20—H20	120.4
C9—C8—C7	116.8 (2)	C19—C20—H20	120.4
C9—C8—H8	121.6	C20—C21—C22	119.1 (2)
C7—C8—H8	121.6	C20—C21—H21	120.4
C8—C9—C10	121.31 (19)	C22—C21—H21	120.4
C8—C9—H9	119.3	C21—C22—C23	120.7 (2)
C10—C9—H9	119.3	C21—C22—H22	119.6
C11—C10—C9	121.80 (19)	C23—C22—H22	119.6
C11—C10—H10	119.1	C22—C23—C18	122.2 (2)
C9—C10—H10	119.1	C22—C23—H23	118.9
C10—C11—C12	118.3 (2)	C18—C23—H23	118.9
C10—C11—H11	120.8	C14—N1—C7	108.51 (15)
C12—C11—H11	120.8	C14—N1—S1	124.51 (13)
C11—C12—C7	118.88 (17)	C7—N1—S1	126.97 (13)
C11—C12—C13	134.02 (18)	O4—N2—O5	123.7 (2)
C7—C12—C13	107.10 (16)	O4—N2—C19	119.0 (2)
C14—C13—C12	107.52 (17)	O5—N2—C19	117.3 (2)
C14—C13—C15	127.04 (17)	O1—S1—O2	121.30 (10)
C12—C13—C15	125.39 (17)	O1—S1—N1	106.80 (9)
C13—C14—N1	109.73 (16)	O2—S1—N1	104.39 (9)
C13—C14—H14	125.1	O1—S1—C1	108.84 (10)
N1—C14—H14	125.1	O2—S1—C1	109.71 (10)
O3—C15—C13	119.40 (19)	N1—S1—C1	104.41 (9)
O3—C15—C16	121.40 (19)		
			
C6—C1—C2—C3	0.3 (3)	C23—C18—C19—C20	2.7 (3)
S1—C1—C2—C3	178.00 (18)	C17—C18—C19—C20	−173.8 (2)
C1—C2—C3—C4	0.2 (4)	C23—C18—C19—N2	−177.95 (19)
C2—C3—C4—C5	−0.3 (4)	C17—C18—C19—N2	5.6 (3)
C3—C4—C5—C6	−0.1 (4)	C18—C19—C20—C21	−3.4 (4)
C4—C5—C6—C1	0.6 (4)	N2—C19—C20—C21	177.3 (2)
C2—C1—C6—C5	−0.7 (3)	C19—C20—C21—C22	1.4 (4)
S1—C1—C6—C5	−178.44 (19)	C20—C21—C22—C23	0.9 (4)
C12—C7—C8—C9	0.6 (3)	C21—C22—C23—C18	−1.5 (4)
N1—C7—C8—C9	179.64 (19)	C19—C18—C23—C22	−0.2 (3)
C7—C8—C9—C10	0.9 (3)	C17—C18—C23—C22	176.3 (2)
C8—C9—C10—C11	−1.0 (3)	C13—C14—N1—C7	−1.3 (2)
C9—C10—C11—C12	−0.6 (3)	C13—C14—N1—S1	178.71 (14)
C10—C11—C12—C7	2.1 (3)	C8—C7—N1—C14	−177.3 (2)
C10—C11—C12—C13	−177.6 (2)	C12—C7—N1—C14	1.8 (2)
C8—C7—C12—C11	−2.2 (3)	C8—C7—N1—S1	2.6 (3)
N1—C7—C12—C11	178.61 (17)	C12—C7—N1—S1	−178.24 (14)
C8—C7—C12—C13	177.62 (18)	C20—C19—N2—O4	−135.8 (2)
N1—C7—C12—C13	−1.6 (2)	C18—C19—N2—O4	44.9 (3)
C11—C12—C13—C14	−179.4 (2)	C20—C19—N2—O5	42.3 (3)
C7—C12—C13—C14	0.8 (2)	C18—C19—N2—O5	−137.1 (2)
C11—C12—C13—C15	3.0 (3)	C14—N1—S1—O1	−139.88 (17)
C7—C12—C13—C15	−176.77 (18)	C7—N1—S1—O1	40.16 (19)
C12—C13—C14—N1	0.3 (2)	C14—N1—S1—O2	−10.27 (19)
C15—C13—C14—N1	177.84 (19)	C7—N1—S1—O2	169.78 (17)
C14—C13—C15—O3	−172.1 (2)	C14—N1—S1—C1	104.91 (17)
C12—C13—C15—O3	5.0 (3)	C7—N1—S1—C1	−75.05 (18)
C14—C13—C15—C16	8.0 (3)	C6—C1—S1—O1	172.14 (17)
C12—C13—C15—C16	−174.90 (18)	C2—C1—S1—O1	−5.6 (2)
O3—C15—C16—C17	−3.1 (3)	C6—C1—S1—O2	37.3 (2)
C13—C15—C16—C17	176.79 (18)	C2—C1—S1—O2	−140.47 (17)
C15—C16—C17—C18	−164.04 (17)	C6—C1—S1—N1	−74.09 (18)
C16—C17—C18—C23	−28.2 (3)	C2—C1—S1—N1	108.15 (18)
C16—C17—C18—C19	148.00 (19)		

### Hydrogen-bond geometry (Å, º)

Cg3 is the centroid of the C7–C12 ring.

**Table:**

*D*—H···*A*	*D*—H	H···*A*	*D*···*A*	*D*—H···*A*
C9—H9···O2^i^	0.93	2.54	3.328 (2)	143
C22—H22···O3^ii^	0.93	2.42	3.319 (3)	163
C23—H23···O5^iii^	0.93	2.36	3.247 (3)	160
C16—H16*A*···*Cg*3^iv^	0.97	2.73	3.565 (2)	144

Symmetry codes: (i) *x*−1, *y*, *z*; (ii) *x*+1, *y*, *z*; (iii) *x*+1/2, −*y*+1/2, *z*+1/2; (iv) −*x*+1, −*y*, −*z*.
