# Supplementary figures and images for: Crystal structure of 3-(2-nitro­phen­yl)-1-(1-phenyl­sulfonyl-1H-indol-3-yl)propan-1-one
Source: Acta Crystallogr E Crystallogr Commun. 2015 Oct 31;71(Pt 11):o892–3. doi: 10.1107/S2056989015020162 (PMC4645035; doi:10.1107/S2056989015020162)

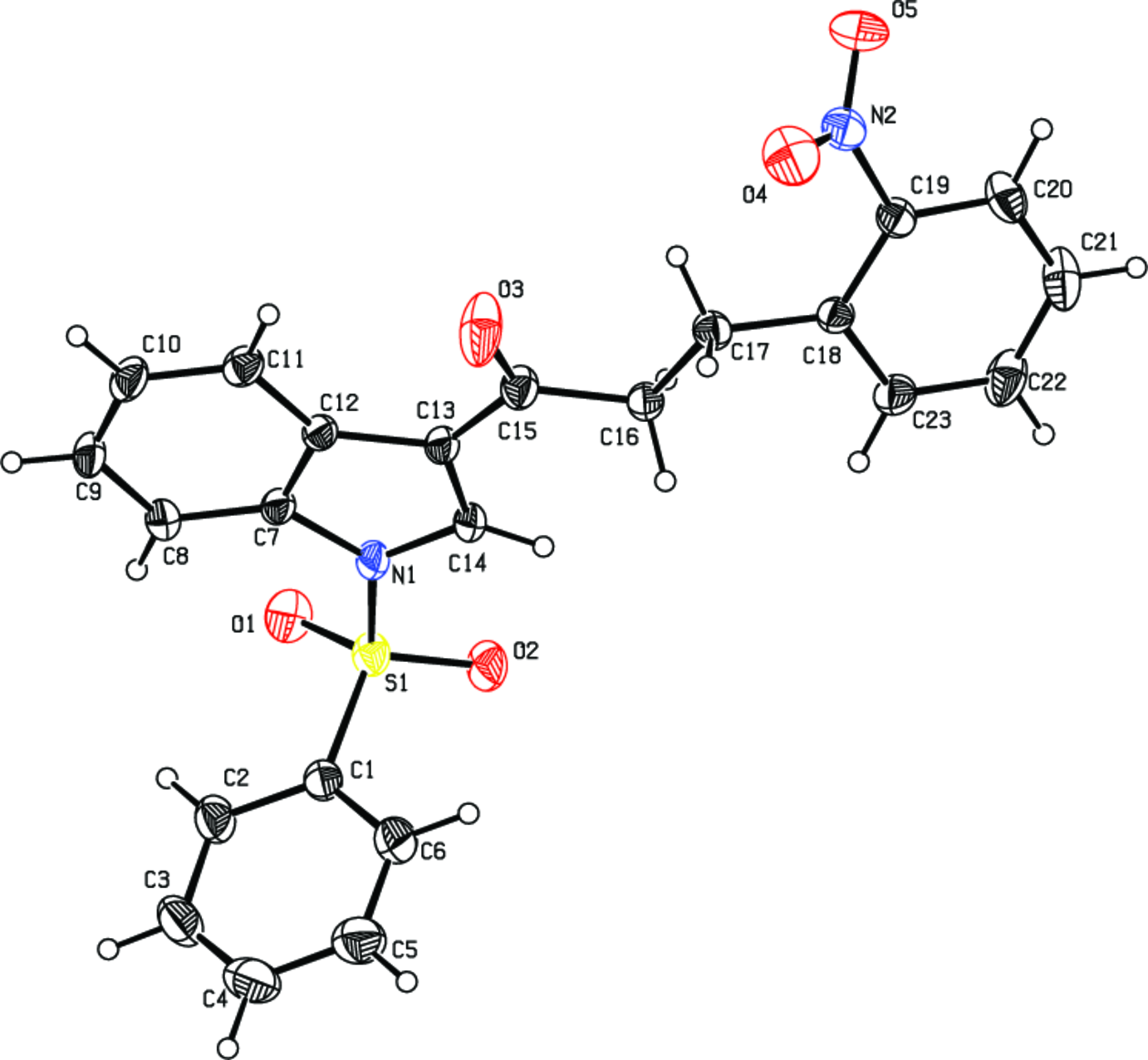

Supplement: Supplementary file 4 [file e-71-0o892-fig1.tif]

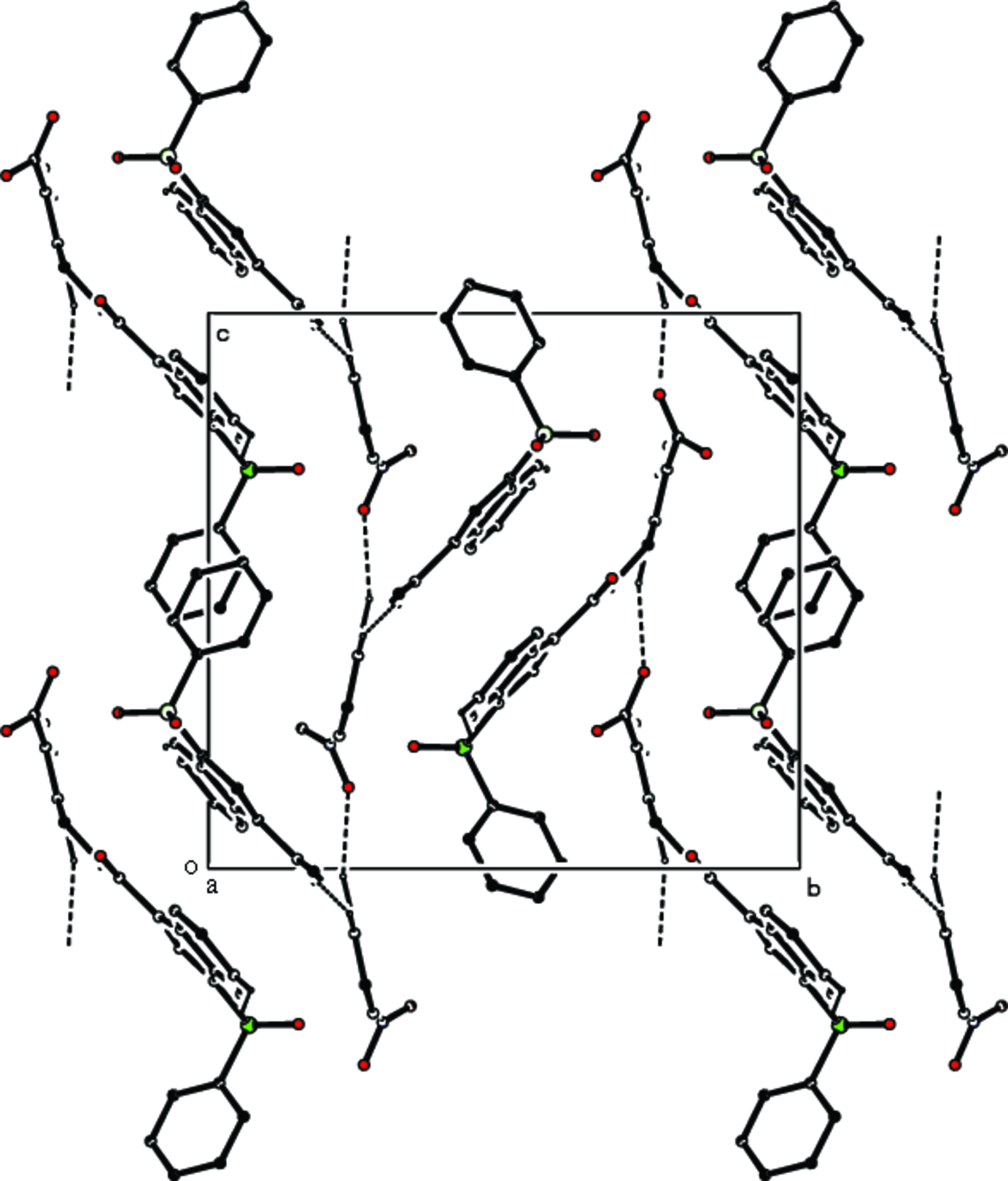

Supplement: Supplementary file 5 [file e-71-0o892-fig2.tif]
